# Influence of Stress, Fatigue, Sleep and Delayed Onset Muscle Soreness on Perceived Physical Enjoyment Exertion during Small Sided Games

**Published:** 2018-03

**Authors:** Okba SELMI, Nejmeddine OUERGHI, Wissam Ben KHALIFA, Nidhal JEBABLI, Moncef FEKI, Anissa BOUASSIDA

**Affiliations:** 1. Research Unit, Sportive Performance and Physical Rehabilitation, High Institute of Sports and Physical Education of Kef, University of Jendouba, Kef, Tunisia; 2. Laboratory of Biochemistry, Rabta Hospital, Faculty of Medicine of Tunis, University of Tunis El Manar, Tunis, Tunisia; 3. Faculty of Sciences of Bizerte, University of Carthage, 7021 Zarzouna, Bizerte, Tunisia

## Dear Editor-in-Chief

In football training, small-sided games (SSG) regimes are generally used as a means to improve players’ technical skills and tactical knowledge. They are more effective and motivating for players than other regimes. Players’ participation in a training exercise corresponds to intrinsic motivation, pleasure, satisfaction, and enjoyment (1). Thus, SSG was produced high physical enjoyment (PE) than high-intensity interval training among youth elite soccer players (1). Indeed, physical and technical responses can be influenced by fatigue accumulation and recovery lack. In this regard, increases in well-being indices during training may reduce performance and cause psychological disturbance among participants (2). Despite the available documents regarding effects of sleep, stress, fatigue, and DOMS on the physiological, technical and psychological responses of a training session (3), no studies have examined the effects of these variables on PE during training exercise. The purpose of this study was to assess the effects of the well-being indices on PE during SSG in soccer players.

During the Tunisian championships season in 2014–2015, there were sixteen U-17 male soccer players competed for the same youth category at a national level took part in the study (mean ± SD: age=16.5±0.6 yr, height=178.2± 64 cm, body mass=684±5.4 kg, body fat percentage=10.6±0.8 and experience in competitive soccer=6.2±1.02 yr). Training data were collected during the last 3 wk of the competitive season. Three training interventions of 4 vs. 4 SSG were performed in separate days and in outdoor field with natural grass on a playing surface of 18 m long and 30 m wide.

The objective of the participants in the training intervention was to maintain a high effort and to keep the possession of the ball for the longest time possible. Coach and physical coach constantly encouraged the participants and quickly provided new balls when needed to maintain the intensity of play until the end of the game. Approximately, 15 min before each training session, each player was asked to rate subjectively the quality of sleep, level of fatigue, stress, and DOMS on a scale of 1–7 (2). Five minutes after training intervention, participants rated how they feel now about the physical activity they have just been doing using Physical Activity Enjoyment Scale (4). Pearson’s correlations were employed to determine the relationships between the variables of interest. Associations of PE with quantity of sleep, stress, level of fatigue and DOMS were not significant (*P*>0.05) (Table 1).

**Table 1:** Correlation of enjoyment with subjective ratings in young soccer players

		***Rating of sleep***	***Rating of stress***	***Rating of fatigue***	***Rating of muscle soreness***	***Hooper index***
**Enjoyment**	R	−0.29	−0.44	−0.37	−0.38	−0.42
	*P*	0.27	0.11	0.15	0.14	0.11

Our results showed that the rating of PE does not seem to be influenced by the variability of the quality of well-being indices during SSG with young players. Stress, fatigue, sleep, and DOMS are not contributing signals to altered PE. Our results may be due to the level of experience of the subjects (all players had been playing football for 4–7 yr) and the choice of exercises realized during the regular training program, including SSG. Well-being indices are used to detect the current form of players, sensitive to the identification of stress after intense training and suitable for monitoring emotional changes of short duration (2). Therefore, the PE can be used as an objective tool for assessing emotional response during training session (5). We consider that motivation factors may clarify a high level of PE. In this context, young football players the most motivated in training session are tose who are the most physically enjoyed (1).

The PE induced by a training method might vary according to modality of exercise, outcomes, and desire of the participants. PE does not seem to be affected by the variability of well-being indices during SSG among young players.
